# Implementing a Low-Cost Non-Destructive Microwave Sensor to Monitor the Real-Time Moisture Content of Rubber Wood in Industrial Dehydration Processes

**DOI:** 10.3390/s25103053

**Published:** 2025-05-12

**Authors:** Thunyawat Limpiti, Charernkiat Pochaiya, Siraporn Sakphrom, Srawouth Chandhaket, Prapan Leekul, Koki Ogura, Tanawut Tantisopharak

**Affiliations:** 1 Center of Excellence Wood and Biomaterials, School of Engineering and Technology, Walailak University, Nakhon Si Thammarat 80160, Thailand; thunyawat.li@wu.ac.th (T.L.); siraporn.sa@wu.ac.th (S.S.); 2 School of Engineering and Technology, Walailak University, Nakhon Si Thammarat 80160, Thailand; pcharern@wu.ac.th (C.P.); csarawou@wu.ac.th (S.C.); 3 Department of Electrical Engineering, Faculty of Industrial Technology, Rambhai Barni Rajabhat University, Chanthaburi 22000, Thailand; prapan.l@rbru.ac.th; 4 Department of Electrical Engineering, Faculty of Science and Engineering, Kyushu Sangyo University, 2-3-1 Matsukadai, Higashi-ku, Fukuoka 813-8503, Japan; ogura.k@ip.kyusan-u.ac.jp; 5 Department of Electrical Engineering, Faculty of Engineering, Khon Kaen University, Khon Kaen 40002, Thailand

**Keywords:** non-destructive measurement, free-space technique, microwave sensor, rubber wood lumber, moisture content monitoring, dehydration process

## Abstract

This study aims to present a low-cost, non-destructive microwave sensor implementation to monitor the real-time moisture content of rubber wood in industrial dehydration processes. The proposed sensor is based on the free-space measurement technique with magnitudes S_11_ and S_21_ only. The novelties of this study consist of the natural frequency determination of rubber wood and the design of a sensor system using devices available on the market with reasonable cost performance. The natural frequency was determined using a simulation and was equal to 1.25 GHz. It specified the sensor system design and device selection. The designed system was initially verified by measuring the moisture content of rubber wood in the laboratory. The measured S_11_ and S_21_ voltages correlating with moisture content percentages were obtained and programmed. The system was then installed to monitor the moisture content of rubber wood in the dehydration process. The measured results deviated from those obtained from a standard method in the range of 7.67–15.38%. The error compensation was analyzed to improve the measured results that provided the deviated moisture content in the range of 3.58–5.21%. It can be inferred that the proposed sensor system has the capability to be implemented in industrial dehydration processes.

## 1. Introduction

Rubberwood is one of the most important agricultural products of Thailand that is locally consumed and exported worldwide. Its demand has been continuously increasing from 2023 to 2024 onwards, especially in China, Korea, and Vietnam; among these three countries, up to 80% of the rubberwood lumber was imported by China from Thailand, with an average volume of 2.7 million cubic meters. The market capitalization of rubberwood lumber is worth 34 billion THB per year in revenue for Thailand [1]. Rubberwood is actually a medium-density hardwood produced by harvesting Para rubber trees (*Hevea brasiliensis*). This lumber is processed prior to being used for industrial products, such as wooden furniture, partition walls, parquet floors, window and door frames, and artworks. The quality control of lumber is essential because moisture content is a major factor affecting it. Typically, the lumber is stored in warehouses with a 12% moisture content level control and must be dehydrated to the moisture content level of 4–8% before use. However, the lumber is quite sensitive to the surrounding environment, as it is prone to attack by insects in a relatively dry environment, while fungal growth is critical when the moisture content is approximately 12–30%. Most industrial plants attempt to maintain the moisture content of lumber at an appropriate level using the dehydration process and monitor the moisture content using a traditional method performed by experts, which involves counting the number of hours spent in the process. This usually results in an inaccurate moisture content level measurement and unnecessary energy expense. The technique of assessing moisture content based on resistance measurements is widely used, but drilling into a lumber sample results in time-consuming sample preparation and a loss of damaged lumber. Thus, a non-destructive moisture content sensor is increasingly needed.

Non-destructive measurements are promising techniques that have been developed because of their advantages, such as being contactless, instantaneous measurement, and the avoidance of sample destruction. For example, the X-ray and magnetic resonance imaging methods provide highly accurate measurements, but their high cost makes them impractical for industrial applications [2]. Other non-destructive measurement methods with moderate accuracy, including ultrasonic, thermographic, and near-infrared, are quite interesting since they are convenient for testing, and this has been consistently reported by many studies [3,4,5,6]. The ultrasonic method determines the moisture content by considering the time, attenuated amplitude, or frequency shift of a transmitted sound wave traveling through the lumber. A higher moisture content results in a lower speed of the ultrasonic wave, which in turn leads to increasing attenuation [7,8,9,10]. However, there are limitations, such as requiring experts in the interpretation of measurement results, expensive maintenance costs, being sensitive to surrounding temperature and humidity, and the need for a smooth and coating-free surface of the sample under testing [11]. The thermographic method detects the emitted infrared radiation (heat) from the surface of an object using thermal cameras to capture thermal inconsistencies due to the moisture content inside the material. The advantage of this technique is its capability of providing a visual map of temperature variation display, but it is significantly affected by surface emissivity and surrounding environmental conditions [12,13,14]. Near-infrared (NIR) spectroscopy analyzes the light absorption or scattering from the lumber correlated with the varied moisture content. This method is suitable for industrial applications since it provides rapid measurement and real-time monitoring capability, but it requires unique calibration for each type of wood, and surface moisture or contaminants strongly affect the accuracy of measurement [15,16,17,18]. According to the aforementioned methods, their performance is limited by the penetration depth, whereby only surface-level moisture is assessed rather than the complete moisture profile of lumber.

A microwave-based technique, particularly the free-space method, is an interesting approach since it allows for rapid and real-time measurements for a bulk of lumber. This method involves transmitting microwave signals through the air (frees pace) into the lumber, then analyzing the attenuation and phase shift of these signals due to the moisture content. It has been widely applied for the measurement of various bulk agricultural products, e.g., paddy, grains, wheat, and peanuts [19,20,21,22,23]. The instrument for industrial timber was first developed at a frequency of 10 GHz, and it was a viable and effective tool with high-precision moisture content measurement and only a deviation of 0.68 [24]. Later, a method was developed to measure not only the moisture content but also the density and grain angle of wood using the attenuation and phase shift of signals at a frequency of 4.81 GHz [25]. In 2001, a method using only reflection and transmission coefficients instead of phase measurements in the frequency range of 8–12.5 GHz was presented [26]. A scanning system with moisture content prediction was developed by using a combination of a microwave camera operating at the frequency of 9.4 GHz and a CT scan [27,28]. In 2009, the attenuation and phase shift measurements at the frequency of the ISM band, i.e., 2.45 GHz, were presented [29]. A high-accuracy method using X-ray for density measurements combined with a microwave method for the attenuation and phase shift measurement at the frequency of 9.375 GHz was proposed [30]. In addition, a practical feasibility study on the free-space method in the industry was performed in the frequency range of 8–12.4 GHz [31]. According to the aforementioned methods, many studies attempted to develop the microwave technique as a practical sensor for use in the industry, but some systems were quite complex due to phase shift measurements. Moreover, some of these systems were expensive as they used a high frequency, i.e., X-ray and CT scan. These systems limited the practical use of these sensors in the industry. In order to solve these problems, the sensor should have two main capabilities: low cost and suitable frequency operation. In terms of low cost, the sensor system can reduce the complexity of measurement by measuring only the magnitude of reflection and transmission signals instead of measuring both the magnitude and phase of signals. For the frequency, it determines the penetration depth inside rubberwood lumber; thus, a suitable frequency should be carefully chosen. The suitable frequency, called natural frequency, provides the best response that can clearly distinguish the moisture content differences.

Consequently, this study presents a low-cost microwave sensor implementation for the moisture content of rubberwood monitoring in the dehydration process. The advantages of this sensor are low cost and the capability of continuously monitoring moisture content in real time. The novel aspects of this study are the determination of the natural frequency of rubberwood using electromagnetic simulation software (v. 2019) [32] and the step-by-step design of sensors using commercially available modules. This article is organized as follows: Section 2 describes Materials and Methods consisting of the sensor system concept and the procedure to determine the natural frequency of rubberwood. The modules used in each part of the system are tested, and the integrated system is then verified for moisture content measurements in the laboratory prior to its installation for the dehydration process. The experimental results are presented in Section 3. The sensor performance is discussed in Section 4. Finally, the conclusions of this study work are provided in Section 5.

## 2. Materials and Methods

The presented microwave sensor is based on the free-space technique, but it differs from the conventional free-space technique that measures the attenuation amplitude and phase shift of signals from the material under test (MUT), which is placed between a transmitting antenna and a receiving antenna. The difference is that the measurement focuses only on magnitudes, the reflection (S_11_) and the transmission (S_21_), since the omission of phase measurement can decrease the complexity of the sensor. In this section, the concept and the operating frequency of the sensor are described.

### 2.1. Concept of a Low-Cost Microwave Sensor

**Figure 1:**
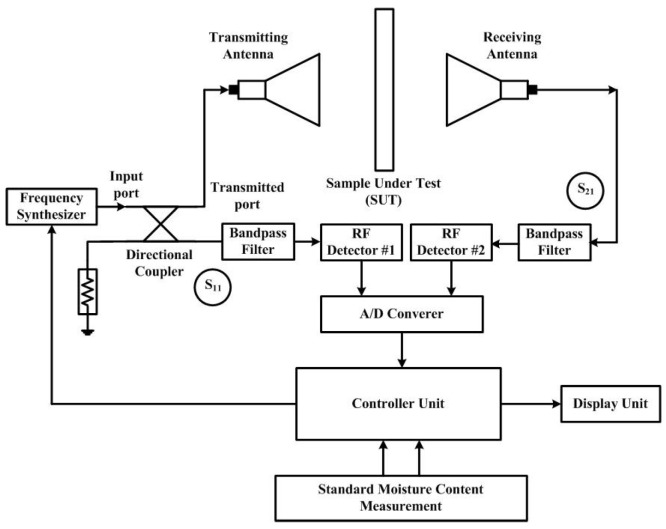
A low-cost microwave sensor diagram.

The sensor is mainly divided into 2 sections, a transmitter and a receiver, as illustrated in Figure 1. The first section consists of a frequency synthesizer, a directional coupler, a bandpass filter, and a transmitting antenna. The latter one is composed of a receiving antenna, a bandpass filter, and a power detector. Both sections are controlled by a microcontroller unit that turns the frequency synthesizer on and programs the operating time for the transmitter section while it receives the measured data to display on the LCD screen and wirelessly sends the data to the cloud for the receiver section.

The concept of this sensor relies on the magnitudes of reflected and transmitted signals from the rubberwood lumber. In the case of S_11_ measurement, the frequency synthesizer generates the frequency and sends it to the transmitting antenna connected to the output port of the directional coupler. The reflected signal comes back to an isolated port of the directional coupler while a coupled port of the directional coupler is terminated with a 50Ω dummy load. For the S_21_ measurement, the receiving antenna receives the transmitted signal through the rubberwood lumber. The magnitudes of both S_11_ and S_21_ are filtered by the bandpass filter prior to converting them to DC voltages with the RF detector #1 and RF detector #2 modules, respectively.

Then, they are converted to digital data in the controller unit to be process with the programmed equation to determine the moisture content of the rubberwood. This equation is the expression of two magnitudes (S_11_, S_21_) related to the moisture content level, the measurements of which are collected from the experiments in the laboratory by varying the moisture content level. These moisture content data are obtained using the ASTM D 4442 standard method [33].

### 2.2. Natural Frequency of Rubber Wood Determination

**Figure 2:**
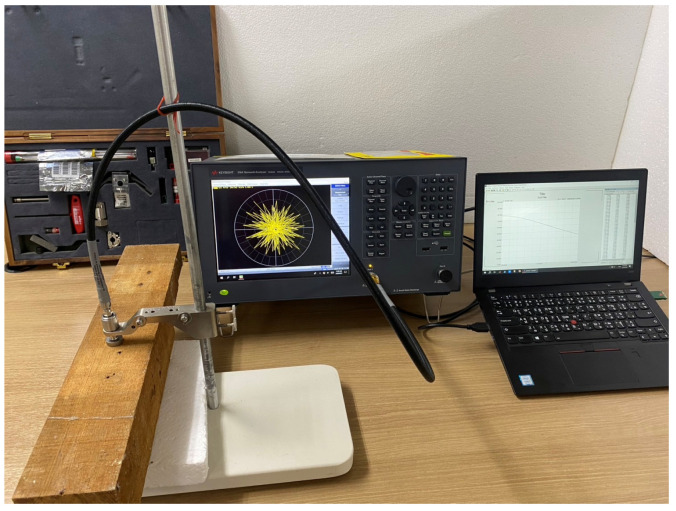
The setup for determining the dielectric properties of rubberwood.

The natural frequency of a material is a frequency at which it noticeably responds to electromagnetic waves with a change in its material properties. These properties are determined with the dielectric constant ($\varepsilon_{r}^{\prime }$) and the dielectric loss factor ($\varepsilon_{r}^{\prime \prime }$). This natural frequency is used as the operating frequency in the microwave sensor systems. In the process of natural frequency determination, the dielectric properties of rubberwood were measured at different moisture content levels using an N1501A dielectric probe connected to an E5063A ENA network analyzer (Keysight, USA), as shown in Figure 2. The measurements were divided into three cases according to the structure of the wood: longitudinal direction, tangential direction, and radial direction. All cases focused on the frequencies in the ISM band, i.e., 0.9, 2.45, 5.8, and 10 GHz; then, the measured data were interpolated using fourth-order polynomial equations for curve fitting the dielectric constant and the dielectric loss factor as a function of moisture content as in [34]. The frequencies below 1 GHz were not considered since the antenna dimension was too large to be practically applied, while the frequencies above 10 GHz were not investigated either because of the high cost of the required equipment. The obtained data, tabulated in Table 1 and Table 2, were then used to define the dielectric constant and the loss tangent ($\varepsilon_{r}^{\prime \prime }/\varepsilon_{r}^{\prime }$) of a model of rubber wood in the commercial electromagnetic simulation software [32].

**Figure 3:**
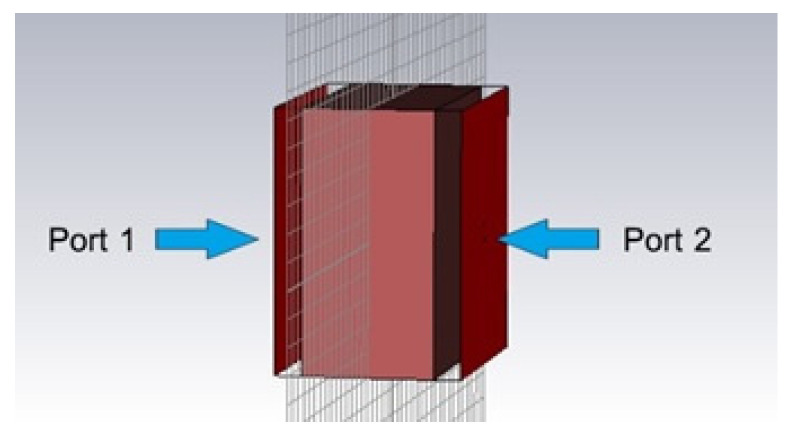
Natural frequency of rubberwood simulation.

**Figure 4:**
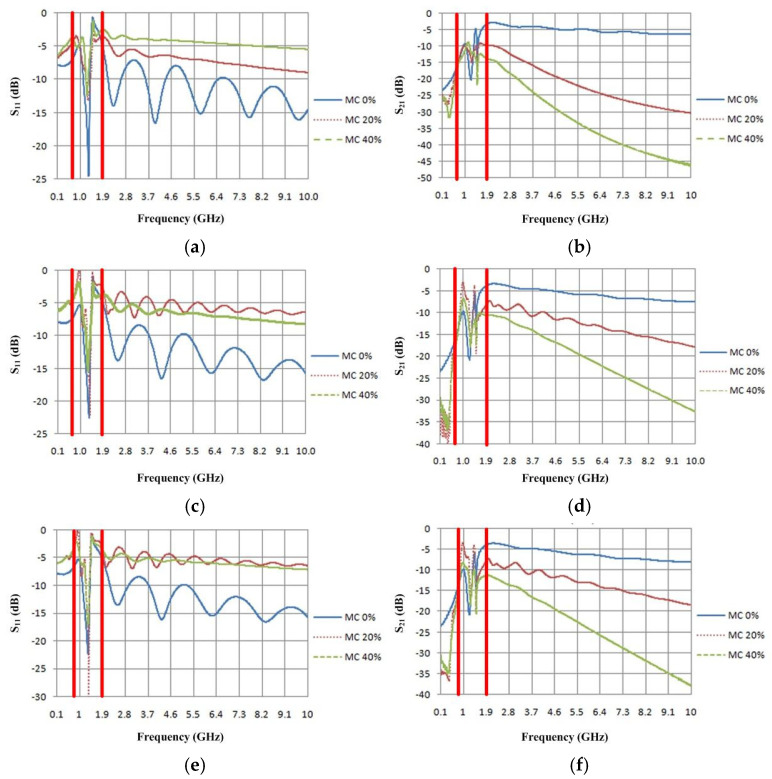
Simulated results: (**a**) longitudinal S_11_, (**b**) longitudinal S_21_, (**c**) tangential S_11_, (**d**) tangential S_21_, (**e**) radial S_11_, and (**f**) radial S_21_.

In the simulation, the rubberwood was modeled at its actual size, where the major parameter was its thickness and depended on the customer demand, i.e., 3 inches and 5 inches. This case used 5-inch-thick rubberwood for the simulation since it was the standard size of the rubberwood that the factory installed in the microwave sensor. The moisture content variations were assessed by changing the dielectric constant and the dielectric loss factor according to the data in Table 1 and Table 2. Then, the simulated results, the reflection coefficient (S_11_) and the transmission coefficient (S_21_), were considered. In order to eliminate the effects of antenna loss and the diffraction at the edge of rubberwood, a pair of waveguide ports was chosen to generate the plane wave as depicted in Figure 3. The simulated results are illustrated in Figure 4. There are three moisture content levels, 0%, 20%, and 40%, that are represented by the blue line, short-dotted red line, and dotted green line, respectively. The frequency span is in the range of 0.1–10 GHz. It can be obviously noticed that the S_11_ results of all cases are matched (S_11_<−10 dB) in a narrow range of frequency, 0.85–1.45 GHz. The frequencies in this range were initially investigated in the S_21_ results to find the frequency that provided the most clearly distinguished value with the different moisture content levels. The trend of S_21_ values decreased as the humidity increased from 20 to 40%. The frequency that showed the most significant difference of approximately 3–5 dB at each humidity level occurred at 1.25 GHz for all cases. Thus, the natural frequency of rubberwood is 1.25 GHz. Notably, the simulations focused on the moisture content range of 0–40% because the moisture content of rubberwood lumber stored in the factory was maintained at approximately 12% and was assessed based on an external moisture of no more than 40%. In addition, this lumber was dehydrated until its moisture content was approximately 4%, since the lumber that was too dry cracked.

## 3. Results

The natural frequency described in the previous section is used to determine the devices to be used in the microwave sensor system integration. In this section, the results are presented in the following sequence: the results of system assembly and testing of the main system components are presented in Section 3.1. Next, the results of the moisture content of rubberwood measured in the laboratory using the microwave sensor system are presented in Section 3.2. Finally, the results of sensor system implementation in the industrial dehydration process and the monitoring of the moisture content of rubber wood are presented in Section 3.3.

### 3.1. Microwave Sensor Integration and Testing

According to the microwave sensor system illustrated in Figure 1, the devices used in each part were investigated from the available modules based on two factors: the natural frequency at 1.25 GHz and low cost. The key parts of the system that define the performance of this system are a frequency generator, a direction controller, a power detector, a bandpass filter, and a pair of transceiver antennas. The selected devices were tested to determine their performance.

#### 3.1.1. Frequency Synthesizer

**Figure 5:**
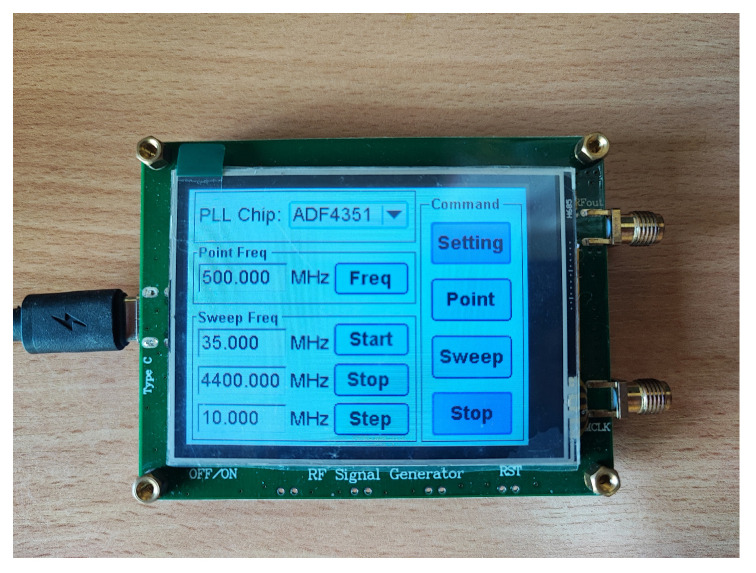
ADF-4351 frequency synthesizer module.

**Figure 6:**
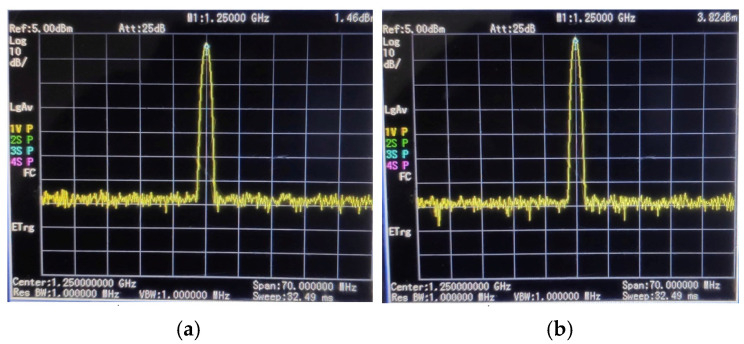
Frequency synthesizer testing: (**a**) +2 dBm output setting and (**b**) +5 dBm output setting.

An ADF-4351 Synthesizer Module is a phase-locked loop RF generator that can generate a stable frequency spectrum and also sweeps the frequency range from 35 MHz to 4.4 GHz. It requires an input voltage of +5 volt DC. The output power can be set at four levels: −4 dBm, −1 dBm, +2 dBm, and +5 dBm. The ADF-4351 module is shown in Figure 5. Initially, it was tested by setting the module to generate a frequency of 1.25 GHz at two output power levels of +2 dBm and +5 dBm. It can be seen in Figure 6 that the output power for these two output levels was +1.46 dBm and +3.82 dBm, respectively. The loss in each output level was 0.54 dBm and 1.18 dBm, respectively.

#### 3.1.2. Directional Coupler

**Figure 7:**
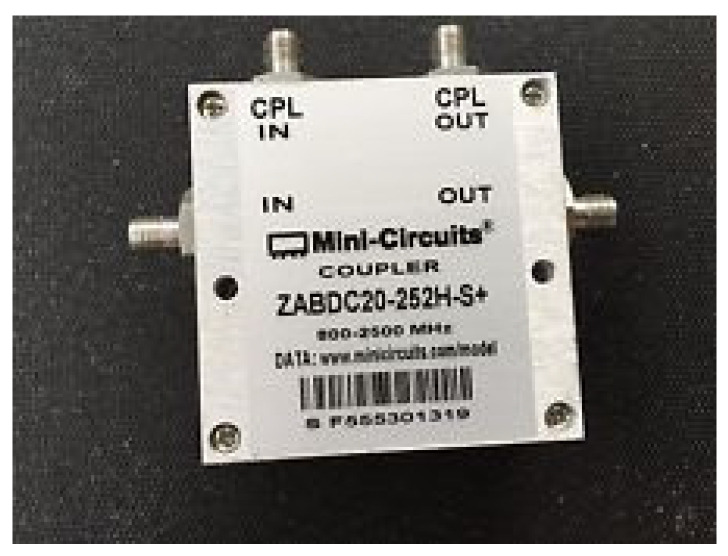
ZABDC20-252H+ directional coupler module.

**Figure 8:**
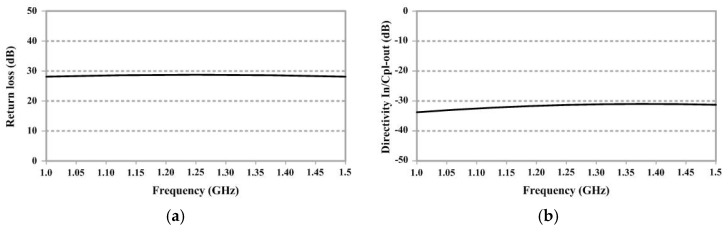
Directional coupler testing: (**a**) return loss and (**b**) directivity input/reverse coupling.

A ZABDC20-252H+ module was chosen to be used as the directional coupler device for detecting the reflection of electromagnetic waves from the rubberwood (S_11_), as shown in Figure 7. This device has four ports: an input port, a couple of in ports, an output port, and a couple of out ports. It can operate in the frequency range of 0.8–2.5 GHz. The basic parameters to characterize the performance of the device were tested by measuring the return loss and the directivity of the input port (IN) to reverse the coupling port (CPL OUT), as shown in Figure 8. Notably, the unused port was terminated with a 50 Ω load. It was found that the return loss and the directivity at the frequency of 1.25 GHz were −29 dB and −33 dB, respectively, as specified in the datasheet.

#### 3.1.3. Power Detector

**Figure 9:**
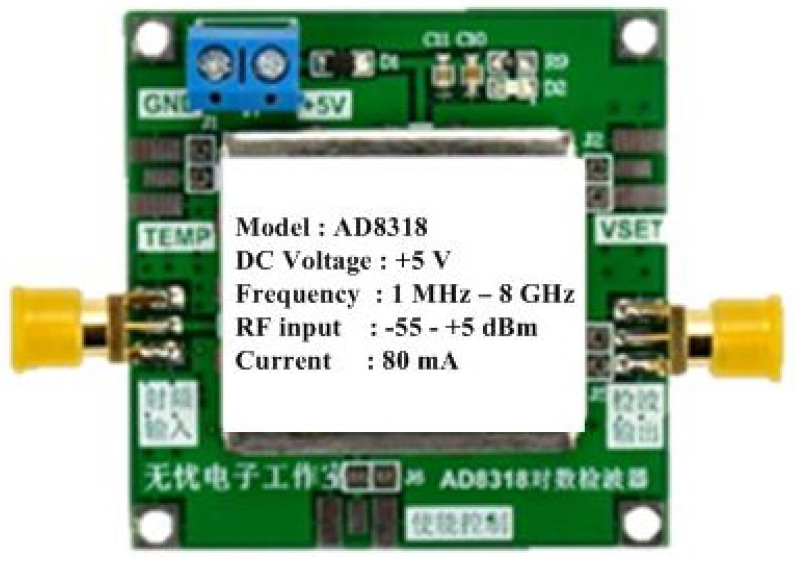
RF power detector module.

**Figure 10:**
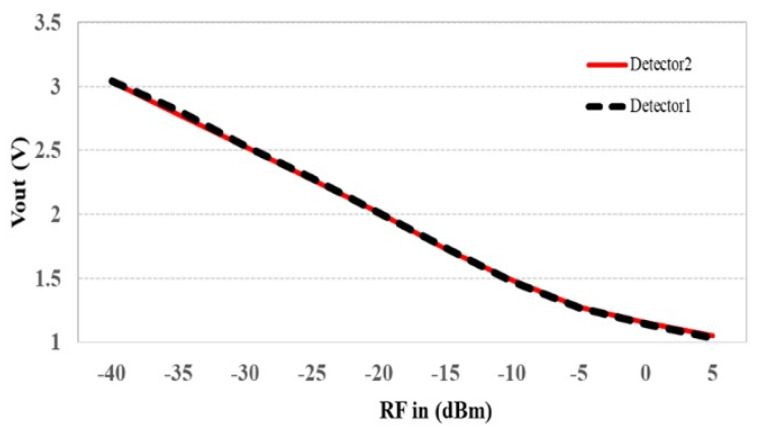
RF power detector testing.

An AD8318 module is a logarithmic power detector that measures the RF power in dBm and converts it to a DC voltage. Its advantage is its capability of measuring power in a wide dynamic range. This module can operate in the frequency range of 1 MHz–8 GHz and can detect the RF power in the range of −55–+5 dBm. It requires an input DC voltage of +5 volts. The AD8318 module is shown in Figure 9. In the microwave sensor system, two power detectors were used to measure S_11_ and S_21_ simultaneously. The devices were tested by feeding an RF signal at 1.25 GHz from a signal generator and varying the power level in the range of −40–+5 dBm. The results are shown in Figure 10. It was found that the output voltage decreased as the RF power increased, and both modules provided identical values for the converted voltage in the range of +3–+1 V, with a resolution of 0.05 V/dB.

#### 3.1.4. Bandpass Filter

**Figure 11:**
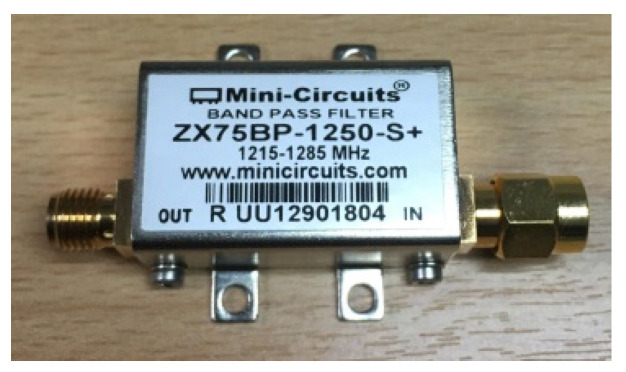
ZX75BP1250-S+ bandpass filter module.

**Figure 12:**
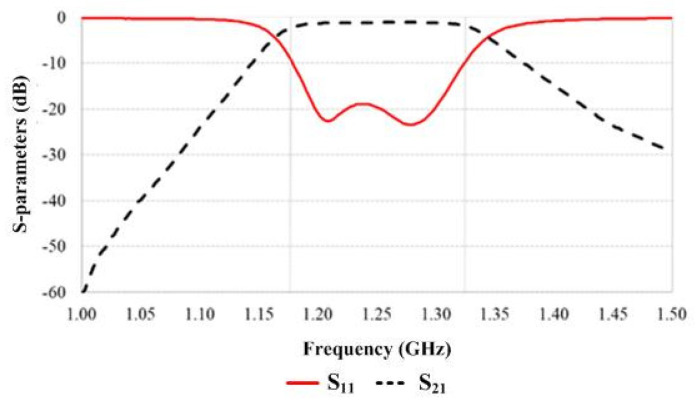
Bandpass filter testing.

A ZX75BP-1250-S+ module was selected to be used as the bandpass filter since the microwave sensor system exhibits a high risk for surrounding electromagnetic interference, such as that caused by the many motors inside the industrial plant and the GSM mobile transmitter (900 MHz) near the industrial plant. This module operates in the frequency range 1215–1285 MHz, as shown in Figure 11. The device was tested by connecting it to an E5063A ENA network analyzer; then, S_11_ and S_21_ were measured. The measured results are shown in Figure 12. It was found that S_11_ was lower than −10 dB in the frequency range of 1.18–1.32 GHz, whereas the insertion loss S_21_ in this frequency range was approximately 2 dB.

#### 3.1.5. Transceiver Antenna

**Figure 13:**
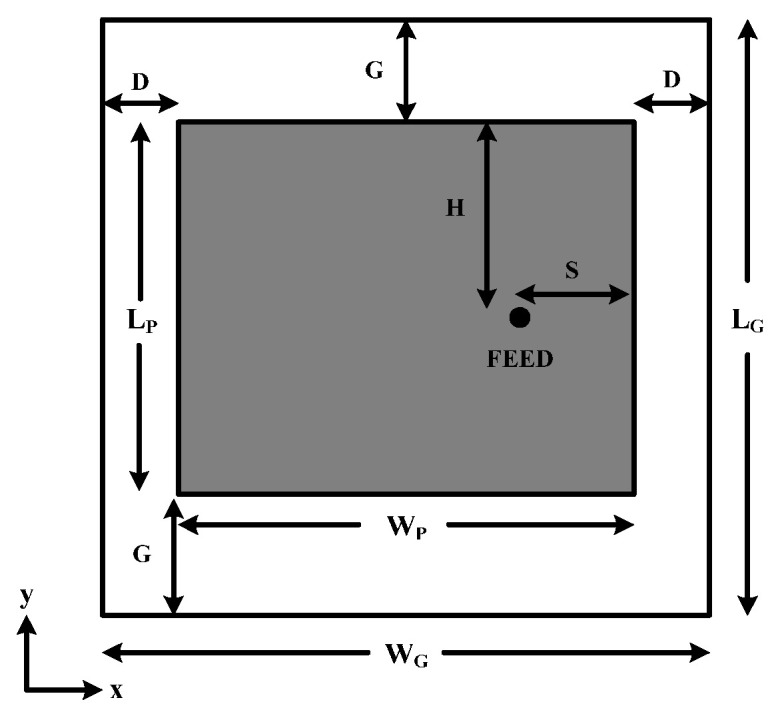
Transceiver antenna structure.

**Figure 14:**
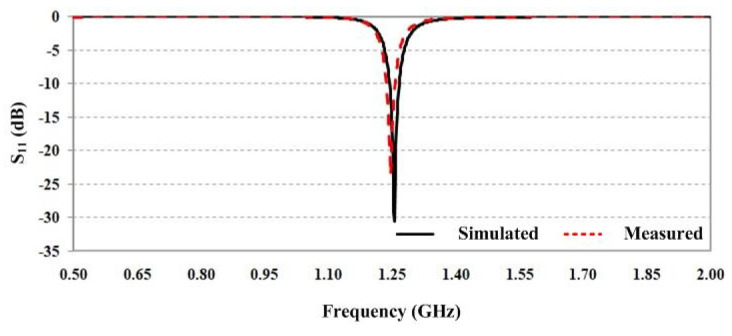
Simulated and measured S_11_ of antenna.

An antenna used as the transmitting/receiving part is a probe-fed square microstrip patch antenna (with a length of $\lambda_{g}/2$, where $\lambda_{g}$ denotes the substrate wavelength) because of its simple shape, compact size, and unidirectional radiation pattern characterization. It was designed on a 1.6 mm thick FR-4 substrate PCB whose dielectric constant and loss tangent were 4.2 and 0.02, respectively, and it was simulated in the electromagnetic simulation software [32] to optimize the dimensions of the antenna at the frequency of 1.25 GHz. The structure of the antenna is shown in Figure 13, and the optimized dimensions of antenna parameters are listed in Table 3. The measured and the simulated reflection coefficient S_11_ at the frequency of 1.25 GHz were −23.65 dB and −29.94 dB, respectively, as illustrated in Figure 14.

**Figure 15:**
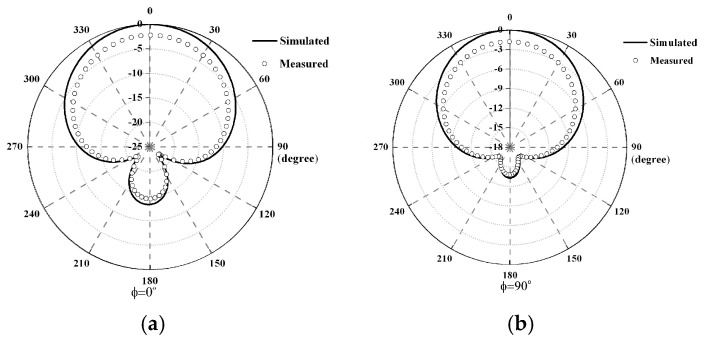
Simulated and measured radiation patterns: (**a**) xz-plane and (**b**) yz-plane.

The simulated and measured radiation patterns are shown in Figure 15. It can be seen that the radiation patterns in both the xz-plane and the yz-plane are symmetrical. The simulated 3 dB beamwidths in the xz-plane and the yz-plane were 91.4 degrees and 92.2 degrees, respectively, while the measured ones were slightly narrower than the simulated results for both the xz-plane and yz-plane. In addition, the antenna gain was 5.52 dBi.

#### 3.1.6. Integrated Microwave Sensor

**Figure 16:**
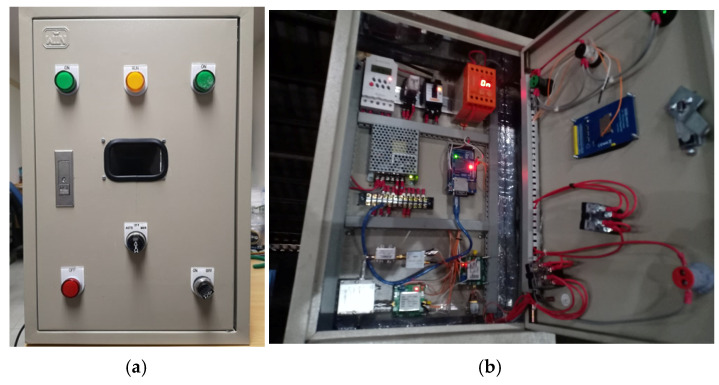
Integrated microwave sensor: (**a**) front panel of cabinet and (**b**) inside of cabinet.

After each part of the microwave sensor system had been tested, they were integrated as depicted in Figure 16. The microwave sensor system has two modes of operation: automatic mode and manual mode. A switch to select the operation mode and two status lights are on the front panel of the control cabinet. A switch on the lower right corner of the control cabinet is used to turn the system on; then, an orange status light is on. The automatic mode is set to measure the moisture content every hour, while the manual mode is a bypass function to simultaneously measure the moisture content. The moisture content status of the rubberwood in the dehydration process is displayed on the LCD screen on the front panel of the control cabinet. In addition, the measured data are backed up on a memory card and are wirelessly sent to be stored in the cloud.

### 3.2. Moisture Content Measurement in Laboratory

In order to verify the performance of the designed microwave sensor system for rubberwood moisture content determination, experiments were initially setup in the laboratory by connecting a transmitting antenna and a receiving antenna with the integrated microwave sensor system, as shown in Figure 16, by using RG58 low-loss coaxial cables, with their characteristics of 50 Ω impedance and 0.558 dB/m attenuation at a frequency of approximately 1 GHz. The sample under test (SUT) was placed between the transmitting and receiving antennas on a sliding rail controlled by a stepping motor to vary the distance between the SUT and antennas. The SUT was rubberwood lumber that had a thickness of 5 inches, and its width and length were three times those of the wavelength to omit the effect of diffraction at the edge. The total number of SUTs was 60 samples, which were cut according to three types of wood structures: longitudinal direction, tangential direction, and radial direction, with 20 samples per type. All samples were assumed to be identical. They were prepared by varying the percentage of moisture content in the range of 7–46% moisture content on the wet basis. The voltage measurement of reflection (S_11_) and transmission (S_21_) was repeatedly performed ten times for each sample inside the anechoic chamber and under a control temperature of 27 °C; then, they were correlated with the moisture content level. The moisture content of SUTs was determined using the ASTM D 4442 standard method, in which the samples were weighed using an electronic balance with an accuracy of 0.001 g prior to heating. They were heated in an oven at a temperature of 130 °C for 19 h; then, they were cooled down in desiccators and weighed. They were repeatedly heated for 6 h and weighed until the change in weight was less than 0.005 g. Finally, the moisture content was calculated from the ratio of the lost weight to the weight before heating.

**Figure 17:**
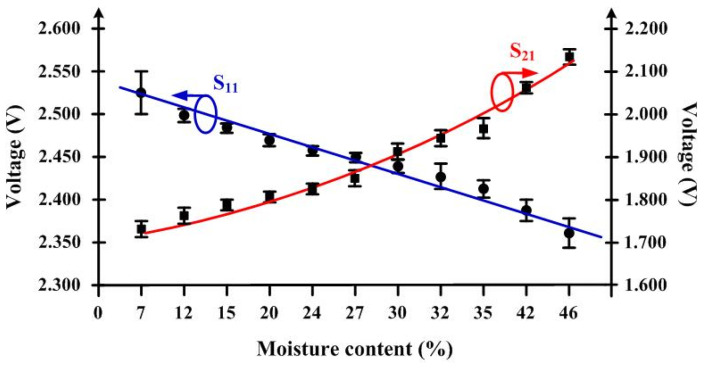
Measured S_11_ and S_21_ voltages versus moisture content.

In the experiments, there were two important variables to be studied: distance and moisture content. Firstly, the S_11_ and S_21_ voltages related to the moisture content were measured, and as shown in Figure 17, each moisture content level showed the maximum, minimum, and average values of voltages. These results were obtained by fixing the distance between the SUTs and antennas under the same conditions as in the simulation, and this distance was equal to 0.75$\lambda_{0}$ for both sides, SUT-to-transmitting antenna and SUT-to-receiving antenna. It is observed that the S_11_ voltage decreases as the moisture content increases and that the voltages are in the range of 2.550–2.345 V. On the other hand, the S_21_ voltage increases in the range of 1.700–2.150 V as the moisture content increases. The S_11_ and S_21_ voltages were curve-fitted to assess the resolution of measurement. The standard deviations in both the voltages and the maximum and minimum values of moisture content obtained from all samples by using the ASTM D 4442 standard method are listed in Table 4.

**Figure 18:**
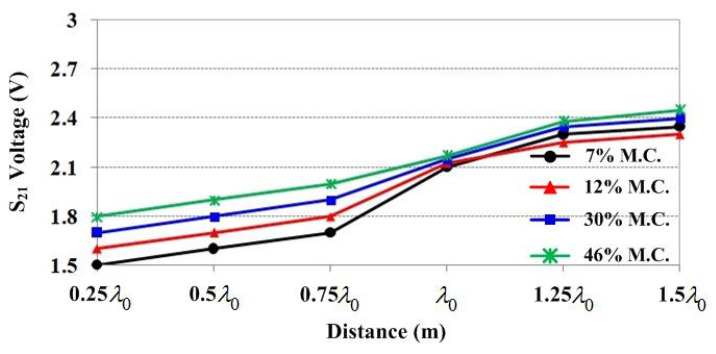
Measured S_21_ voltage versus moisture content for distance variation.

The effect of the distance between SUTs and antennas was studied next. In this case, the SUT-to-transmitting antenna and SUT-to-receiving antenna distances were set to be equal and varied from 0.25$\lambda_{0}$ to 1.5$\lambda_{0}$. At each distance, there were four moisture content levels: 7%, 12%, 30%, and 46%. The measured S_21_ voltage results are illustrated in Figure 18. It can be seen that they increase as the distance increases, and they are distinguishable at the distance range of 0.25$\lambda_{0}$–0.75$\lambda_{0}$, but they are so close that it is hard to distinguish the moisture content at a distance more than $\lambda_{0}$ due to the insufficient sensitivity of the RF power detector module. Therefore, to apply this system in the industry, an RF power amplifier is required to provide sufficient power for the RF power detector for efficient detection.

### 3.3. Moisture Content Monitoring in Dehydration Process

**Figure 19:**
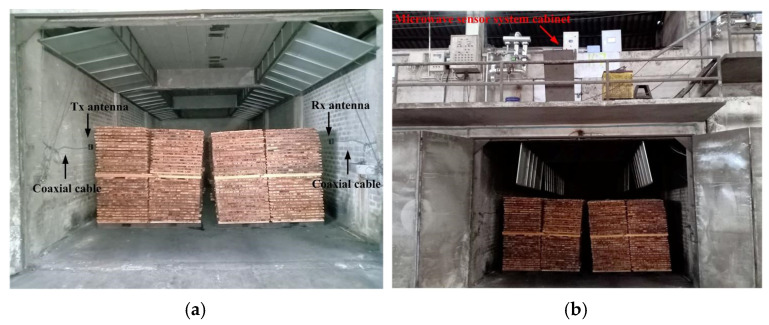
Microwave sensor system installation in dehydration process: (**a**) inside and (**b**) outside.

According to the experiments in the previous subsection, the RF power is an essential factor that allows the microwave sensor system to effectively detect moisture content; when the distance between rubberwood lumber and antennas in the dehydration process is more than $\lambda_{0}$, the RF power is too weak to be detected. Thus, the link budget concerning the scenario of microwave sensor system installation needs to calculate the provision of sufficient RF power. The interior and exterior scenarios of the heating room are shown in Figure 19. The transmitting antenna and receiving antenna are mounted on opposite sides of the concrete walls inside the heating room. These antennas are shielded inside domes to prevent their performance degradation due to the moisture content at the feeding points and the temperature effect. An increase in temperature increases the dielectric constant and affects the dielectric loss factor of antenna substrates [35]. It shifts the resonant frequency to a higher frequency.

**Figure 20:**
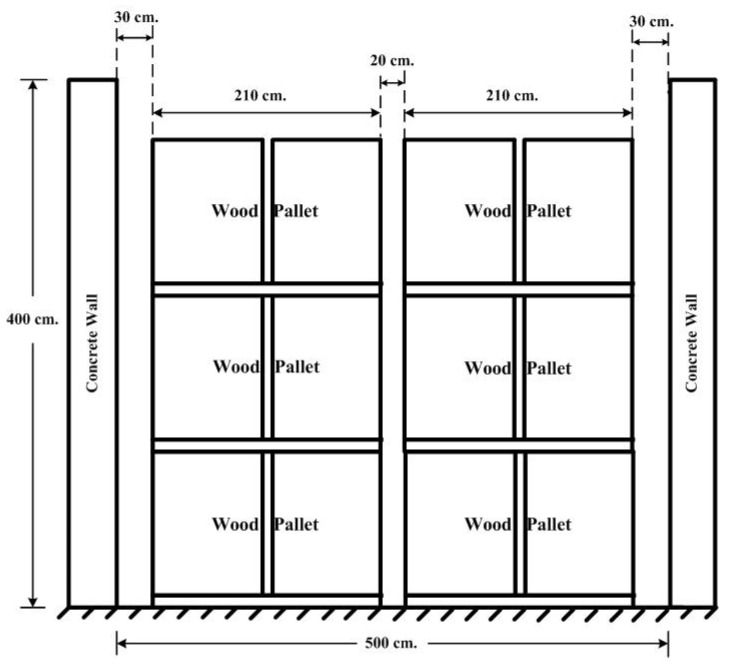
Heating room construction and its dimensions.

The microwave’s RF power is generated for the transmitting antenna through a 12 m RG58 coaxial cable, which is shielded inside a heat-resistant conduit from the microwave sensor system cabinet mounted outside the heating room. The receiving antenna is also connected to the microwave sensor system cabinet with a 12 m RG58 coaxial cable on the opposite wall. The heating room construction and its dimensions are illustrated in Figure 20. Its width and height are 5 m and 4 m, respectively, and the rubberwood lumber is placed on wood pallets that are arranged in two rows with a 3 m height, a 2.1 m width, and a 0.2 m separation. In addition, they are 0.3 m away from the side wall. The transmitting RF power can be calculated from the Friis formula as follows:

(1) $$P_{r}=P_{t}+G_{r}+G_{t}+20\log\left(\frac{\lambda }{4\pi d}\right)$$

where $P_{r}$, $P_{t}$, $G_{r}$, $G_{t}$, λ, and d denote receiving power (dBm), transmitting power (dBm), gain of transmitting antenna (dBi), gain of receiving antenna (dBi), wavelength (m), and the separation distance between transmitting and receiving antennas (m). In the calculation, the transmitting antenna and receiving antenna have an identical gain of 5.52 dBi, and the range of receiving power was specified using the RF power detector performance, which was in the range from −40 dBm to −5 dBm. The calculated result showed that the transmitting power was +32.32 dBm. Moreover, the attenuation loss in the RG88 coaxial cable was 0.558 dB/m. Thus, we chose the RF power amplifier with a gain above +45 dB for use.

**Figure 21:**
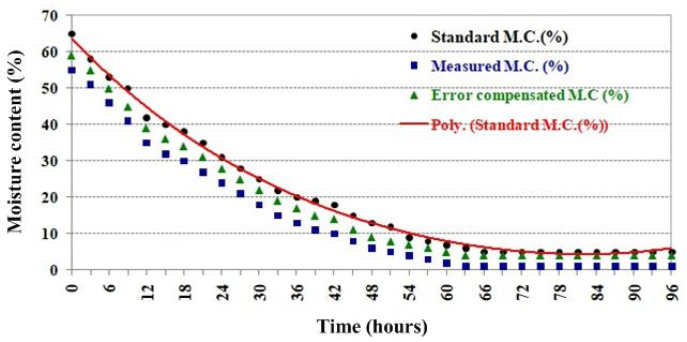
Moisture content monitoring during the dehydration process.

In the conventional dehydration process, the rubberwood lumber was continuously heated at a constant temperature of 80 °C for 96 h inside the heating room. In order to validate the microwave sensor system, the S_11_ and S_21_ voltages were measured; then, these results were processed with the processor that had been programmed with the equation correlating both voltages with the moisture content level obtained from the experiments in the laboratory. The moisture content level was displayed on the LCD screen in front of the microwave sensor system cabinet. The measurement was performed every 3 h. The measured results compared with the moisture content obtained from the standard moisture meter installed inside the heating room are shown in Figure 21.

The measured moisture content results from the proposed microwave sensor system are represented as blue squares, while the standard moisture content values are represented as black circle dots. The red line is curve-fitted to the standard moisture content values to show the trend of moisture content. The moisture content of the dehydration process started at 64%. Notably, the moisture content rapidly decreases at 48 h, and after that, it gradually decreases in the saturation phase. The measured results are lower than the standard results at all moisture content levels. These deviated moisture content results are in the range of 7.67–15.38%. The reason for these deviated results is the different conditions of SUTs for the experiments in the laboratory and the experiments of the dehydration process. Moreover, an increase in temperature degrades the performance of antennas by shifting the operating frequency, which causes the magnitude of both S_11_ and S_21_ to reduce. Thus, the measured moisture content is lower than the actual moisture content. Furthermore, the temperature effect is inevitable, and it can only be reduced by shielding.

### 3.4. Error Compensation

**Figure 22:**
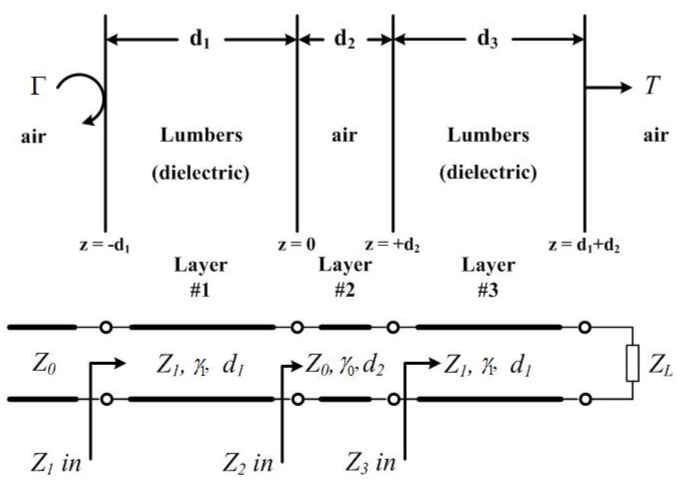
Transmission line model of multilayer structure for error compensation.

Due to the deviations in the measured moisture content results, the error compensation had to be analyzed and corrected. This error was caused by the inhomogeneous SUT medium, resulting in multiple reflections and mismatches at the discontinuity boundaries. The conventional boundary condition of free-space measurement has three layers, i.e., air–dielectric–air, but the boundary condition in this case has five layers, i.e., air–dielectric–air–dielectric–air, as illustrated in Figure 22. Thus, the moisture content results were lower than the actual values. A generalized transmission line model (TLM) for a multilayer structure [36,37] was applied to analyze this problem. In the analysis, the rubberwood lumber was modeled as lossy transmission lines with an electrical length of $\gamma_{1}d_{1}$ and a characteristic impedance of $Z_{1}$, while $\gamma_{0}d_{2}$ and $Z_{0}$ were the electrical length and characteristic impedance for the middle, where γ represented the propagation constant. It was terminated by a load impedance of $Z_{L}$, which was the air backing behind the rubberwood lumber. The air layer in front of the rubberwood lumber was represented by an infinite length transmission line with a characteristic impedance of $Z_{0}$.

The input impedances of the equivalent transmission lines that are connected in tandem can be iteratively calculated as follows:

(2) $$Z_{in}=Z_{1}\left(in\right)=Z_{1}\left(\frac{Z_{2}\left(in\right)+Z_{1}\tanh\left(\gamma_{1}d_{1}\right)}{Z_{1}+Z_{2}\left(in\right)\tanh\left(\gamma_{1}d_{1}\right)}\right)$$

(3) $$Z_{2}\left(in\right)=Z_{0}\left(\frac{Z_{1}\left(in\right)+Z_{0}\tanh\left(\gamma_{01}d_{2}\right)}{Z_{0}+Z_{1}\left(in\right)\tanh\left(\gamma_{0}d_{2}\right)}\right)$$

(4) $$Z_{3}\left(in\right)=Z_{3}\left(\frac{Z_{L}+Z_{3}\tanh\left(\gamma_{1}d_{3}\right)}{Z_{3}+Z_{L}\tanh\left(\gamma_{1}d_{3}\right)}\right)$$

The transmission coefficients from the first incident medium #0 (air) through a multilayer structure to the last medium #*N* + 1 (air) can be calculated using the following formula:

(5) $$T=\left(n_{N+1}/n_{0}\right){\left|\frac{I_{N+1}}{I_{0}}\right|}^{2}$$

where

(6) $$I_{N+1}=I_{1}\overset{N}{\underset{i=1}{\Pi }}\left[\cosh\left(\gamma_{i}d_{i}\right)+\frac{Z_{i+1}(in)}{Z_{i}}\sinh\left(\gamma_{i}d_{i}\right)\right]$$

The parameters n, $I_{0}$, $I_{i}$, and $I_{N+1}$ denote the reflective index, the normal incident electric field, the input current of layer #i, and the input current of the last layer, respectively.

The reflection and transmission coefficients obtained from the error compensation analysis were used as multiplier factors to calibrate the measured reflection and transmission coefficient values prior to converting them to the moisture content. The compensated moisture content results were plotted as green triangles, as shown in Figure 21. Evidently, the error in the moisture content results was lower in the range of 3.58–5.21% compared to that in the standard results.

## 4. Discussion

Based on the results in the previous sections, it is worth discussing the limitations and the accuracy of the proposed microwave sensor system. At first, the main goal of this study was to implement a low-cost sensor system for practical usage in the industry; thus, all devices or modules were chosen based on their being of a commercial grade with a reasonable price. The dynamic range of the RF power detector module is an essential factor in determining the sensitivity of detection, i.e., this study uses the logarithmic RF power detector that can detect the power in the range of −5–+5 dBm, with a resolution of 0.05 V/dB. When the dimensions of the heating room and the distance of antennas to the rubberwood lumber increase, the receiving power decreases till it is undetectable. Thus, the size of the heating room is related to the RF power detection that should be sufficiently provided by the link budget management.

The transmitting and receiving antenna installation inside the heating room is also an important factor since a misaligned installation causes the RF power to become lower than the actual value. A pair of microstrip patch antennas with a gain of 5.51 dBi were designed for use in this study. The 3 dB radiation pattern of this antenna covers 46.3 degrees. In the experiments, the misalignment of antennas was offset by more than 50 degrees, resulting in power reduction to approximately 12 dB. A solution for this problem may be carefully installing other high-gain antennas, which have structures suitable for installation on the sidewall without causing obstruction.

In addition, the error compensation of the measured moisture content using the transmission line model analysis can improve accuracy by reducing the error in the ranges of 7.67–15.38% and 3.58–5.21%. According to Figure 21, the dehydration process is programmed to continuously maintain a constant temperature of 80 °C for 96 h. The sensor system continuously monitors the moisture content, and it is observed that the moisture content reaches a saturation point when the dehydration time reaches 70 h. This process should be finished early, i.e., within 26 h, to reduce the waste of energy and save energy. In the future, accuracy enhancement should be performed by employing antennas with high gains and narrow beamwidths to focus the radiated power on the rubberwood. A circularly polarized antenna is also a good candidate for reducing interferences. In addition, increasing the number of sensors and employing AI can aid in decision-making.

## 5. Conclusions

This study presents the implementation of a low-cost, non-destructive microwave sensor for monitoring the moisture content of rubberwood in real-time in the industrial dehydration process. The proposed sensor system only employed the magnitudes of S_11_ and S_21_. Firstly, the natural frequency of rubberwood was determined using a simulation, and it was equal to 1.25 GHz. It was used to specify the frequency of sensor system operation. Each part of the sensor system was tested to characterize the performance of devices prior to integrating them into the system. In order to verify the designed sensor system, experiments were set up to measure the moisture content of rubberwood in the laboratory. The measured S_11_ and S_21_ voltages were then correlated with the moisture content percentages using the ASTM D 4442 standard method. The sensor system was installed to monitor the moisture content of rubberwood in the dehydration process for 96 h. The measured results were compared to those obtained from the standard method, and they deviated in the range of 7.67–15.38%. Thus, the error compensation was analyzed to improve the measured results. The deviation in the compensated moisture content result decreases in the range of 3.58–5.21%. In addition, the dehydration time reached a saturation phase in 70 h based on the monitoring of the proposed sensor system; however, the time required to reach a saturation phase should be reduced to almost 26 h for reducing energy wastage and saving energy costs. The proposed sensor system can be implemented for moisture content monitoring in industrial dehydration processes.

## Figures and Tables

**Table 1:** Measured dielectric constant of rubberwood.

	Longitudinal				Tangential				Radial			
	0.9 GHz	2.45 GHz	5.8 GHz	10 GHz	0.9 GHz	2.45 GHz	5.8 GHz	10 GHz	0.9 GHz	2.45 GHz	5.8 GHz	10 GHz
**0% M.C.**	2.75	2.68	2.49	2.23	2.28	2.17	2.13	1.94	2.34	2.24	2.13	1.98
**10% M.C.**	3.87	3.74	3.69	3.36	4.05	3.72	3.17	3.00	3.88	3.32	3.06	3.69
**20% M.C.**	6.97	5.83	5.17	4.02	5.66	4.49	4.42	3.43	5.89	5.18	4.66	3.69
**30% M.C.**	10.88	9.56	8.30	6.86	6.31	5.61	4.94	4.26	8.43	7.27	6.36	5.48
**40% M.C.**	15.35	13.39	12.58	8.97	7.38	6.63	5.63	4.47	9.17	8.43	7.35	6.3
**50% M.C.**	16.70	11.90	10.80	9.83	8.34	7.56	6.67	5.70	12.12	10.78	9.14	8.05
**60% M.C.**	18.00	16.40	13.40	11.00	10.13	8.87	8.10	6.30	13.09	12.42	11.22	9.29
**70% M.C.**	18.40	16.90	14.10	11.80	10.80	9.92	9.08	2.79	15.10	14.00	12.60	11.50
**80% M.C.**	19.90	18.60	15.40	12.80	13.00	12.00	10.80	9.23	20.50	19.00	16.40	14.30

**Table 2:** Measured dielectric loss factor of rubberwood.

	Longitudinal				Tangential				Radial			
	0.9 GHz	2.45 GHz	5.8 GHz	10 GHz	0.9 GHz	2.45 GHz	5.8 GHz	10 GHz	0.9 GHz	2.45 GHz	5.8 GHz	10 GHz
**0% M.C.**	0.21	0.21	0.22	0.23	0.10	0.21	0.25	0.28	0.18	0.23	0.27	0.35
**10% M.C.**	0.47	0.53	0.65	0.73	0.32	0.39	0.42	0.54	0.37	0.41	0.49	0.59
**20% M.C.**	1.24	1.49	1.75	1.79	0.71	0.81	0.97	1.18	0.65	0.92	1.03	1.19
**30% M.C.**	1.93	2.34	3.08	3.12	0.86	1.26	1.29	1.45	0.94	1.35	1.70	1.97
**40% M.C.**	2.57	3.15	4.07	4.34	1.00	1.38	1.61	1.82	1.17	1.68	2.08	2.36
**50% M.C.**	2.84	3.60	4.94	5.23	1.11	1.45	2.00	2.27	1.40	2.14	2.86	3.10
**60% M.C.**	2.97	3.80	5.49	5.83	1.29	1.78	2.51	2.79	1.99	2.38	3.50	3.85
**70% M.C.**	3.05	3.96	5.93	6.41	1.44	1.80	2.76	3.16	2.28	3.52	3.93	4.74
**80% M.C.**	3.22	4.14	6.62	6.97	1.78	2.26	3.24	3.82	3.09	4.10	5.34	6.01

**Table 3:** Antenna parameters and their optimized dimensions.

Parameters:	*W_G_*	*L_G_*	*W_P_*	*L_P_*	*D*	*G*	*H*	*S*
**Dimension: (mm)**	114	114	56.2	56.2	28.9	28.9	28.1	14.05

**Table 4:** Measured voltage deviation versus moisture content variation.

Moisture Content (%)	Measured Voltage (V)		Moisture Content Variation (%)	
	S_11_	S_21_	Minimum	Maximum
7	2.525 ± 0.025	1.725 ± 0.025	7.05	7.56
12	2.500 ± 0.006	1.760 ± 0.015	11.84	12.70
15	2.475 ± 0.005	1.780 ± 0.020	14.92	15.83
20	2.465 ± 0.005	1.800 ± 0.020	19.46	21.20
24	2.460 ± 0.007	1.825 ± 0.020	23.55	24.90
27	2.450 ± 0.005	1.850 ± 0.025	26.27	27.85
30	2.435 ± 0.008	1.900 ± 0.025	29.78	30.31
32	2.425 ± 0.016	1.940 ± 0.025	31.64	32.40
35	2.400 ± 0.010	1.955 ± 0.035	34.23	36.21
42	2.388 ± 0.012	2.060 ± 0.020	41.70	42.89
46	2.360 ± 0.015	2.125 ± 0.025	45.58	46.74

## Data Availability

All data are shared in this paper.
